# Mechanical Properties’ Strengthening of Photosensitive 3D Resin in Lithography Technology Using Acrylated Natural Rubber

**DOI:** 10.3390/polym15204110

**Published:** 2023-10-17

**Authors:** Wasan Tessanan, Philippe Daniel, Pranee Phinyocheep

**Affiliations:** 1 Department of Chemistry, Faculty of Science, Mahidol University, Rama VI Road, Payathai, Bangkok 10400, Thailand; t.wasan18@gmail.com; 2 Institut des Molécules et des Matériaux du Mans (IMMM), UMR CNRS 6283, Faculté des Sciences et Technologie, Le Mans Université, Bd O. Messiaen, CEDEX 09, 72085 Le Mans, France; philippe.daniel@univ-lemans.fr

**Keywords:** natural rubber, modified natural rubber, acrylated natural rubber, bio-based material, digital light 3D printing processing

## Abstract

Acrylated natural rubber (ANR) with various acrylate contents (0.0–3.5 mol%) was prepared from natural rubber as a raw material and then incorporated with commercial 3D resin to fabricate specimens using digital light processing. As a result, the utilization of ANR with 1.5 mol% acrylate content could provide the maximum improvement in stretchability and impact strength, approximately 155% and 221%, respectively, over using pure 3D resin, without significant deterioration of tensile modulus and mechanical strength. According to evidence from a scanning electron microscope, this might be due to the partial interaction between the dispersed small rubber particles and the resin matrix. Additionally, the glass-transition temperature of the 3D-printed sample shifted to a lower temperature by introducing a higher acrylate content in the ANR. Therefore, this work might offer a practical way to effectively enhance the properties of the fundamental commercial 3D resin and broaden its applications. It also makes it possible to use natural rubber as a bio-based material in light-based 3D printing.

## 1. Introduction

Over the past few decades, three-dimensional (3D) printing, an additive manufacturing (AM) process, has been widely used in a variety of industry verticals, particularly in the electronic, pharmaceutical, automotive, aerospace and defense, and food sectors [1,2,3,4]. It has the potential to fabricate the physical 3D parts from a computer-aided design (CAD) model by assembling them layer by layer [5,6,7,8]. The advantages of 3D printing technology over conventional subtractive manufacturing (SM) processes include the ability to create objects that are free to be customized, have complex shapes, and produce less waste. Therefore, this technology has been considered as a disruptive and transformative process using various materials [9,10,11]. Digital light processing (DLP), a VAT photopolymerization-based 3D printing process, has drawn more attention than other types of 3D printing technology because of its exceptional features, which include comfortable working conditions, low energy consumption, fast printing, and high printing accuracy [12]. The DLP-based 3D printing system uses a digital light projection of an ultraviolet (UV) curing system to photopolymerize liquid photosensitive resin into a solid polymer in a spatially controlled manner [13,14].

The majority of commercially available photosensitive resins for DLP-based 3D printing typically have an acrylate base and solidify via the free radical photopolymerization process [15,16]. The substance used in 3D printing is a thermosetting polymer. The thermoset polymeric material’s mechanical performance is closely correlated with its molecular architecture, which can be modified by a number of photocuring system-dependent parameters (such as monomer composition, photoinitiator content, curing conditions, and optical light source) [17,18]. Generally, the acrylate-based resin has a much higher rate constant for radical polymerization (12.64 ± 0.41 × 10^3^ (s^−1^) for ethyl acrylate) than methacrylate type (2.84 ± 0.28 × 10^3^ (s^−1^) for ethyl methacrylate), which likely results in the inhomogeneous molecular architecture of the crosslinking reaction. The photocured product consequently suffers from the problem of high volumetric shrinkage and brittleness [19,20]. Additionally, the liquid photosensitive 3D resin’s extensive properties, such as its mechanical and thermal properties, are still insufficient for end-use applications [15,20,21]. Consequently, the issue with liquid resin has been the subject of extensive research. Various conventional procedures have been exploited for the tunability of the thermomechanical properties of the desired 3D resin, including designing the resin formulation, chemically altering the constituents, and adding mechanical modifiers or additives [18,22,23]. The latter procedure, using polymer blends and composite materials, is of enormous interest due to the ease of the process, cost-effectiveness, and high efficacy.

Generally, rubber or elastomeric material can be utilized as a toughening or mechanical modifier for brittle polymers, and thus natural rubber (NR) has been used for this purpose [24,25]. NR is known as a green elastomer and a renewable polymer consisting mainly of *cis* 1,4-polyisoprene. It is obtained from tapping the *Hevea brasiliensis* tree in the form of NR latex as a colloidal rubber particle suspension [26,27]. The well-known aspects of NR have been revealed, including low cost, high elasticity, high abundance, renewability, and easy chemical modification [28]. Various types of chemical modification can be carried out in the latex stage as a colloidal system as well as an organic solution using one, two, or even three steps of modification depending on the intended applications.

For example, epoxidation is a one-step chemical modification, while epoxidized natural rubber, having low molecular weight, was carried out using an epoxidation reaction followed by an oxidative degradation reaction [29]. Furthermore, photosensitive materials, obtained from natural rubber and developed through chemical modifications for exploitation in surface coating application, were reported on [30,31] and adhesive and sealing applications [32]. As far as our investigation is concerned, there have only been a few reports on the use of natural rubber and its derivatives as a mechanical modifier to enhance the properties of acrylate-based resin for 3D printing based on lithographic technology. As demonstrated in our previous work [33], photosensitive natural rubber (PNR) was synthesized via the chemical modification pathways using natural rubber latex as a raw material. The PNR, having a low molecular weight (M_n_ of 4700 g/mol) and acrylate content (0.4 mol%), was obtained and utilized as a mechanical modifier for blending with the commercially available photocurable 3D resin. The rubber/3D resin blend with different rubber content (0.9–3.0 wt.%) was formulated and patterned the specimen using the DLP-type 3D printer. As a result, the printed materials made with photosensitive rubber (containing 0.4 mol% acrylate) may be more ductile and impact-resistant than those made with neat 3D resin without the addition of rubber. Nevertheless, the final conversion, the modulus, and the mechanical strength all decreased simultaneously due to the presence of PNR. Therefore, it is still challenging to find a suitable acrylate content that can be attached to the NR chain in lithography 3D printing technology and improve printed specimen’s toughness without noticeably degrading tensile modulus and mechanical strength.

Consequently, we aim to synthesize photosensitive rubbers with various acrylate contents (0.0–3.5 mol%) from NR latex as a raw material. The modified rubbers were then blended with the commercial 3D resin and subjected to the 3D printing process. The mechanical, morphological, and thermal characteristics of the printed specimen, as well as the chemical interaction of the rubber and resin matrix, were studied in order to find out more about the radical photopolymerization of the lithographic 3D printing process.

It is advantageous as a substitute strategy for enhancing the rubber/3D resin blend system. As a result of the study, it may be beneficial to use natural rubber, a green elastomer, as a photosensitive mechanical modifier in light-based 3D printing technology.

## 2. Materials and Methods

### 2.1. Materials

High ammonia NR latex (HANR, 60% dried rubber content, DRC) was purchased from Thai Rubber Latex Co., Ltd. (Chonburi, Thailand). Tergitol 15-S-15 (non-ionic surfactant) was obtained from Sigma Aldrich (St. Louis, MI, USA). Hydrogen peroxide (35%) and ammonium hydroxide solution (25%) were purchased from QRec Chemical Co., Ltd. (Auckland, New Zealand). Formic acid (85%) was supplied by Carlo Erba Reagent (Milan, Italy). Acrylic acid (99%) was ordered from Shandong Sparrow Chemical Co., Ltd. (Jinan City, China). Periodic acid (99%) was purchased from Shanghai Runwu Chemical Technology Co., Ltd. (Shanghai, China). ANYCUBIC 3D printing UV-sensitive resin was procured from Shenzhen Anycubic Technology Co., Ltd. (Shenzhen, China). According to the safety data sheet (SDS) of this typically commercial 3D resin, it is an acrylate-based resin consisting of isooctyl acrylate (10–40%), polyurethane acrylate (30–60%), and photoinitiator (2–5%).

### 2.2. Synthesis of Acrylated Natural Rubber (ANR)

**Figure 1:**
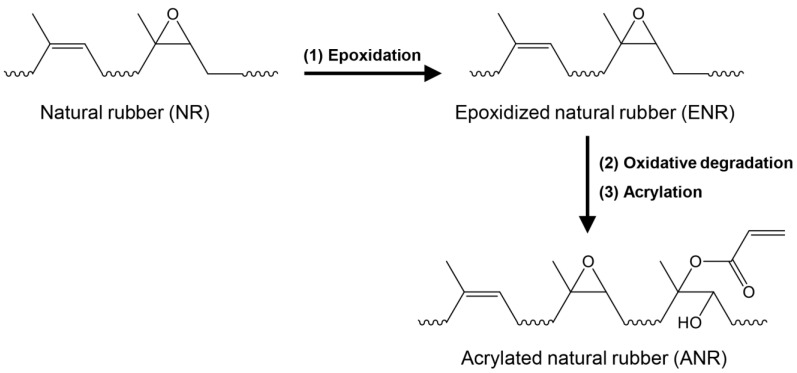
Schematic preparation of acrylated natural rubber using three-step chemical modification.

ANR was synthesized from natural rubber latex via three steps of chemical modifications, as illustrated in Figure 1. We prepared 20% DRC of NR latex by diluting HANR latex (60% DRC) with distilled water before stabilizing with 3 parts per hundred rubber (phr) of tergitol 15-S-15 and stirring for 24 h. Firstly, in-situ performic acid, obtained by adding 0.25 mol of formic acid and 1 mol of hydrogen peroxide into the prepared natural rubber latex, was carried out for epoxidation reaction at 60 °C for 3 h, resulting in epoxidized natural rubber (ENR) latex. After cooling down, the prepared ENR latex was brought to room temperature, the pH of the latex was adjusted to 7 by adding ammonium hydroxide solution, before secondly performing oxidative degradation using 0.5 mol of periodic acid at 40 °C for 12 h, in order to lower the molecular weight of the ENR. The latex was then coagulated in methanol and washed with excess water before drying at 40 °C in a vacuum oven. At the third step of chemical modification, 20 g of the prepared low molecular weight ENR was dissolved in 300 mL of toluene before the acrylation reaction using different amount of acrylic acid (3, 6, and 9 mol) at 75 °C for 12 h. The obtained acrylated NR (ANR) solution was coagulated with methanol before washing with water several times. The coagulated ANR was dried in the vacuum oven at room temperature (25 ± 2 °C) for seven days. The ANR was then analyzed and the sample code was defined as AxNR, where x is the mol% of acrylate content attached to the NR backbone; for instance, A0.4NR means ANR containing 0.4 mol% of acrylate moieties.

### 2.3. Fabrication of 3D Printing Part

#### 2.3.1. Preparation of ANR/3D Resin Blend

The prepared ANRs with various acrylate contents were dissolved in toluene to prepare the rubber solutions (30% *w*/*w*). Afterwards, 5 wt.% of the prepared rubber solutions (1.5 wt.% ANR content) were blended with 95 wt.% of the commercial 3D resin with stirring until a homogeneous solution was obtained (1 h). Subsequently, the blended resin was sonicated in an ultrasonicator (50 W and 40 kHz) in the fume hood for 30 min to evaporate the toluene solvent and well disperse the ANR in the 3D resin. Ultimately, some bubbles and the toluene residual were removed from the blend resin for 3 min at room temperature using a vacuum oven. The blending formulation is summarized in Table 1.

#### 2.3.2. Preparation of 3D Printing Object

All formulations of the ANR/3D resin blend were patterned into the tensile and impact specimens using a DLP-type 3D printer (SK-US01, Sparkmarker, Hong Kong, China). Firstly, the 3D object models with the typical STL (Standard Triangle Language) file format of tensile and impact specimens with the sizes conforming to ASTM D638 [34] and ASTM D256 [35], respectively, were built using Autodesk 123D Design software (v2.2.14). After that, the designed 3D models were sliced to a 50 µm thickness for 3D printing in each layer using SparkStudio software (v2.0), a slicer program of Sparkmarker.

**Figure 2:**
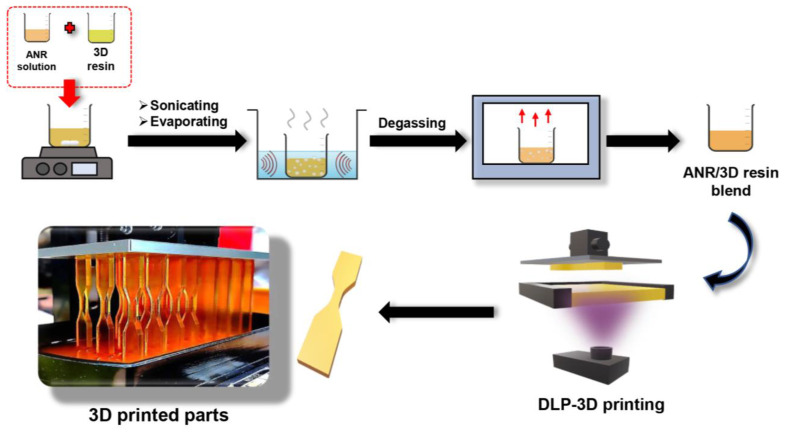
Schematic preparation of the ANR/3D resin blend and the 3D printing process.

Secondly, in the 3D printing procedure, the blended resin (50 g) was filled into the resin tray before involving the printing task. Subsequently, the 3D-patterned specimens were taken off the printing platform, followed by cleaning to remove debris and residue resin form the samples with isopropanol. Ultimately, the post-processing step was performed using UV irradiation (60 W, λ = 405 nm) for 30 min at room temperature in the dark box to obtain the complete curing product. The schematic preparation of the ANR/3D resin blend and the 3D-printed part is illustrated in Figure 2.

### 2.4. Characterization

#### 2.4.1. Chemical Structure Analysis

The attenuated total reflection equipped with Fourier transform infrared (ATR-FTIR) spectra were obtained from the infrared spectrophotometer (Paragon 1000, PerkinElmer, Waltham, MA, USA). The measurement was carried out with a resolution of 4 cm^−1^ and 64 scans by scanning from 4000 to 400 cm^−1^.

The proton nuclear magnetic resonance (^1^H-NMR) spectra were obtained by operating a nuclear magnetic resonance spectrometer with frequency of 500 MHz (Bruker Corporation, Karlsruhe, Germany). Deuterated chloroform (CDCl_3_) was employed as a solvent, and tetramethylsilane (TMS) was used as an internal standard. Moreover, the acrylate content of the prepared ANR was determined by the integration of the signal area using Equation (1) [33].

Acrylate content (mol%) = (A_6.42_/(A_6.42_ + A_5.10_ + A_2.70_)) × 100 (1)

where A_2.70_, A_5.10_, and A_6.42_ are the integrated signal area of methine protons near the epoxide ring, methine protons adjacent to the double bond of the isoprene unit, and methine protons of the acrylate functional groups, respectively.

#### 2.4.2. Molecular Weight Analysis

The molecular weight of the modified natural rubbers was determined using gel permeation chromatography (GPC) (150-C ALC/GPC, Waters, Milford, MA, USA). Approximately 10 mg of rubber samples were dissolved in tetrahydrofuran (THF) as a solvent and filtered through a nylon membrane filter (22 µm pore size) before analysis. THF as an eluent was manipulated at 40 °C with 1 mL/min of a constant flow rate. The number average molecular weight (M_n_), the weight average molecular weight (M_w_), and the polydispersity index (PDI) of the rubber samples were obtained in this measurement.

#### 2.4.3. Rheological Measurement

The rheological manner and viscosity of the blended resin were conducted using a coaxial cylinder rheometer. (Physica MCR 500, Anton Paar, Graz, Austria). The measurement was performed at room temperature with shear rate between 0.1 s^−1^ and 100 s^−1^ to obtain the viscosity data as a function of the shear rate.

#### 2.4.4. Insoluble Content and Photopolymerization Kinetics

The insoluble content or gel content related to the crosslinking density of the 3D-printed sample was evaluated using the Soxhlet extraction method using an excess of toluene solvent at 130 °C for 24 h. The insoluble fraction was dried in an oven until a constant weight was obtained, and the gel content was determined, conforming to Equation (2).

Gel content (%) = (*w*/*w_o_*) × 100 (2)

where *w_o_* and *w* are the dry weight of the sample before and after the Soxhlet extraction, respectively.

Furthermore, the soluble fraction was precipitated in cold methanol (0 ± 2 °C). The precipitated rubber, which was referred to as unreacted rubber of ANR in the sol content after Soxhlet extraction, was dried in a vacuum oven at 60 °C until a constant weight was acquired. Three times for measurements were performed to obtain the mean value and standard deviation. Finally, the reacted rubber content could be calculated using Equation (3).

Reacted rubber content (%) = ((*w_i_* − *w_p_*)/*w_i_*) × 100 (3)

where *w_i_* is the dry weight of rubber in the printed sample before a Soxhlet extraction, and *w_p_* is the dry weight of precipitated rubber in the sol content after Soxhlet extraction.

The double bond conversion (DBC) involving the photopolymerization kinetic profiles was measured using ATR-FTIR analysis. The cured thin sheets (100 ± 5 µm) with different cure times (0–600 s) of each formulation were fabricated in the dark box using the UV curing process (light intensity = 200 mW·cm^−2^ and λ = 405 nm). The DBC was received by the integrating of the absorption peak area of the acrylate double bond (1403 cm^−1^). Meanwhile, the integrated peak area of the carbonyl group at 1728 cm^−1^ was used as an internal standard, and the calculation of DBC was determined using Equation (4) [36].

DBC (%) = 1 − ((A_1403_/A_1728_)_t_/(A_1403_/A_1728_)_o_) × 100 (4)

where (A_1403_/A_1728_)_o_ is the peak area ratio between the double bond of acrylate and the carbonyl groups before the UV curing process. (A_1403_/A_1728_)_t_ is the peak area ratio between the double bond of acrylate and the carbonyl groups at different times of the UV curing process. 

Moreover, the photopolymerization rate (R_p_) was also determined using Equation (5) [37].

R_p_ = d(DBC)/dt (5)

where d(DBC)/dt represents the derivative of DBC with respect to time.

#### 2.4.5. Mechanical Test

The tensile performance of the 3D-patterned sample was measured using a universal tensile testing machine (Instron 5566, Instron, High Wycombe, UK). Eight specimens of each 3D-printed sample were conducted according to ASTM D638 using a crosshead speed of 10 mm/min and a static load cell capacity of 1 kN at room temperature (25 ± 2 °C). Tensile properties, including Young’s modulus (E), tensile strength (σ), and elongation at break (ɛ), were acquired in this measurement.

Impact properties related to the fracture resistance of the printed sample were investigated according to ASTM D256 by an impact tester machine (5102 Pendulum, Zwick, Ulm, Germany). Six specimens were performed with Izod mode under room temperature and reported in the mean value in the unit of kJ/m^2^.

#### 2.4.6. Morphological Analysis

The phase morphological aspect of the printed sample was observed from the impact-fractured surface using a scanning electron microscope (SEM) (Hitachi SU 8000, Hitachi, Ibaraki, Japan). Before the observation, the samples were coated with platinum/palladium (Pt/Pd, 80/20) to prevent electrostatic charge accumulation.

#### 2.4.7. Thermal Analysis

The printed sample’s glass transition temperature (T_g_) was evaluated using a differential scanning calorimeter (DSC) (Perkin Elmer DSC-7, Perkin Elmer, Waltham, MA, USA). Approximately 10 mg of each printed sample was weighed and put in the aluminum pan before heating from 0 to 120 °C with a constant heating rate of 10 °C/min under a heat–cool–heat cycle. 

## 3. Results and Discussion

### 3.1. Chemical Structure and Molecular Weight Analysis of Acrylated Natural Rubber

**Figure 3:**
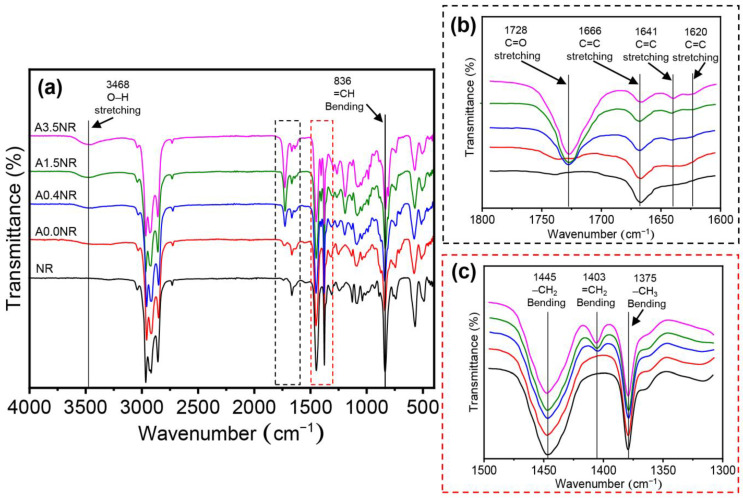
ATR-FTIR spectra of the modified natural rubber at various ranges of wavenumber: (**a**) 4000–400 cm^−1^, (**b**) 1800–1600 cm^−1^, and (**c**) 1500–1300 cm^−1^.

The intricate chemical structure of the acrylated natural rubber (ANR) was conducted using attenuated total reflection Fourier transform infrared (ATR-FTIR) and proton nuclear magnetic resonance (^1^H-NMR) analysis. Figure 3 illustrates the ATR-FTIR spectra of the prepared ANR at various acrylate contents (A0.0NR, A0.5NR, A1.5NR, and A3.5NR). The absorption spectrum of NR as a raw material showed the double bond peaks of the isoprene unit at 836 cm^−1^ (=CH bending) and 1666 cm^−1^ (C=C stretching). Other absorption peaks are the C-H stretching vibration (2800–3000 cm^−1^) and C-H bending vibration (1375 and 1445 cm^−1^) [33,38]. After the three-step chemical modifications to incorporate acrylate moieties to the NR backbone, the prepared ANRs exhibited the absorption peak of the acrylate double bond at 1403, 1620, and 1641 cm^−1^ [30,39]. Moreover, the absorption peaks of the carbonyl group (1728 cm^−1^) and the hydroxyl group (3468 cm^−1^) were also detected in the molecular structure of the prepared ANR [33,40].

**Figure 4:**
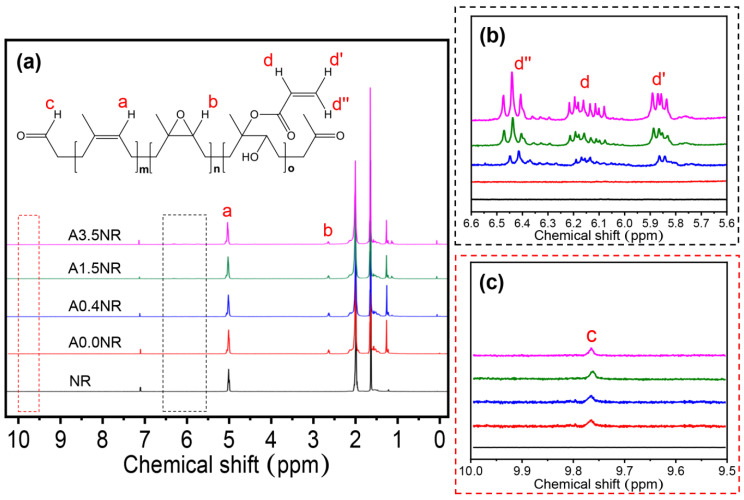
^1^H-NMR spectra of the modified natural rubber at various ranges of the chemical shift: (**a**) 0–10 ppm, (**b**) 5.6–6.6 ppm, and (**c**) 9.5–10.0 ppm.

Subsequently, the ^1^H-NMR analysis was utilized to ensure the intricate chemical structure of the synthesized ANR, as shown in Figure 4. The NR as a starting material revealed the characteristic signal at 5.10 ppm (a) corresponding to the methine proton of the isoprene unit. After the chemical modifications, the prepared ANRs displayed the signals of methine proton adjacent to the epoxide functional group (b) at 2.70 ppm and protons of acrylate functional group (d, d′, and d″) at 6.18, 5.86, and 6.42 ppm, respectively (Figure 4b) [33]. In addition, the small intensity signal of the methine proton of the aldehyde functional group (c) at 9.78 ppm, obtained from the oxidative degradation step, was detected, as demonstrated in Figure 4c [29].

**Figure 5:**
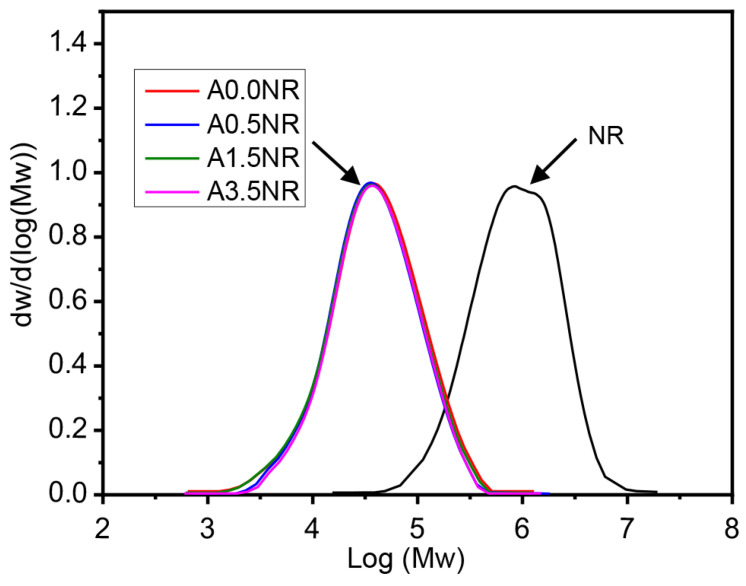
Molecular weight and polydispersity index of NR and the modified NRs.

Therefore, the ATR-FTIR and ^1^H-NMR analysis confirmed the successful attachment of acrylic acid as a photosensitive molecule on the NR backbone through the self-acid catalyzed epoxide ring-opening process of the acrylation reaction. Furthermore, the acrylate content of the prepared ANR was determined using the integrated peak area of the ^1^H-NMR spectrum following Equation (1). The calculated result showed the prepared ANR having 0.42, 1.52, and 3.51 mol% of acrylate content, which is defined as A0.4NR, A1.5NR, and A3.5NR, respectively. The A0.0NR was referred to the modified NR to ENR without acrylate content. The molecular weight of the prepared ANRs was also determined using gel permeation chromatography (GPC), as shown in Figure 5, and the relevant value can be summarized in Table 2. As a result, the number average molecular weight (M_n_) and the weight average molecular weight (M_w_) of the raw material NR were approximately 900,000 g/mol and 1,200,000 g/mol, respectively. The epoxidation through an in situ performic acid system, followed by an oxidative degradation reaction using periodic acid as an oxidant, could provide a lowering of the molecular weight of the modified NR (L-ENR) or A0.0NR having M_n_ and M_w_ about 14,000 g/mol and 47,000 g/mol, respectively, with the PDI of 3.26. After the acrylation reaction to attach different amounts of photosensitive acrylate group, the prepared ANRs possessed M_n_ and M_w_ at approximately 14,500 g/mol and 46,000 g/mol, respectively, with a PDI of ~3.20. Consequently, the attachment of the small amount of acrylate molecule to the rubber backbone had no significant impact on the molecular weight of the prepared ANR. The key rationale for lowering the molecular-weight of the NR is contributing to the miscibility between the rubber-dispersed phase and the liquid 3D photosensitive resin during the 3D-printing-based lithographic process.

### 3.2. The 3D-Printed Part of the Rubber/3D Resin Blend

#### 3.2.1. Rheological Characterization and Viscosity Measurement 

**Figure 6:**
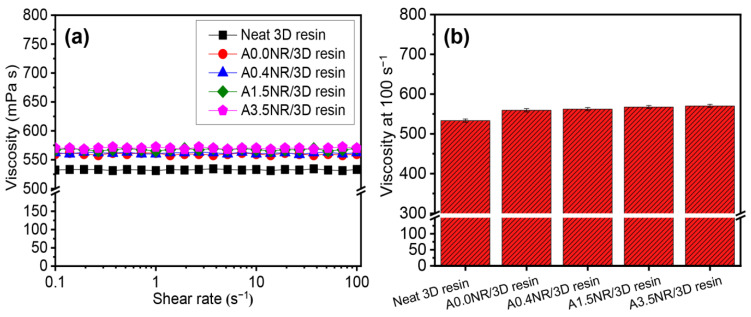
Rheological properties of the ANR/3D resin blend with various acrylate contents of ANR at the same rubber ratio: (**a**) rheological manner and (**b**) viscosity at 100 s^−1^.

The rheological fashion of the ANR/3D resin blends, with various acrylate content of ANR in the liquid form, was assessed under the shear rate of 0–100 s^−1^ at room temperature (25 ± 2 °C) using a rheometer, as illustrated in Figure 6. As a result, the neat commercial 3D resin showed Newtonian behavior throughout the given shear rate. Thus, this could elucidate that the viscosity of the neat commercial 3D resin was not dependent on the shear force [41]. Furthermore, the rheological aspect was not affected by the addition of ANR; however, there was a slight increment in the viscosity of the ANR/3D resin blend system (Figure 6a). Generally, the viscosity of the mixture resin was firstly considered as it is vital for printability. It was reported that the high viscosity of the resin could restrict the solidification process of the objects during the printing step [42,43]. The viscosity value of the light-based 3D printing resin was usually reported at a high shear rate of 100 s^−1^ [44]. As seen in Figure 6b, the viscosity value of the neat commercial 3D resin was approximately 533 mPa·s. The addition of 5% ANR solution (1.5 wt.% ANR) affected a slight escalation of viscosity compared to the neat resin. Additionally, the viscosity value increased gradually from 559 to 570 mPa·s when the acrylate content of ANR increased from 0 to 3.5 mol%. This phenomenon could be ascribed to the higher number of acrylate pendant groups along the molecular structure of modified rubber, which might promote the chain entanglement or restrict the chain motion, causing a higher resistance to the flow of the liquid resin under the rheological test. 

#### 3.2.2. Photopolymerization 

**Figure 7:**
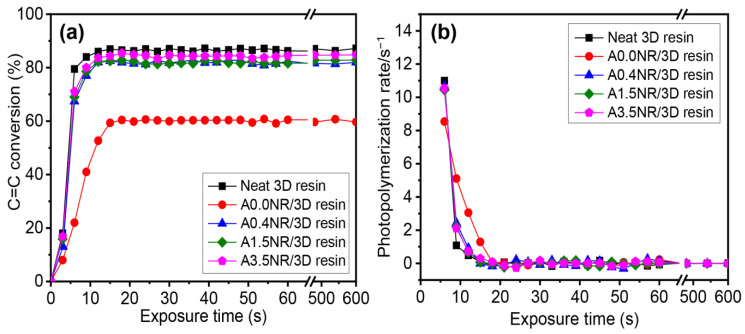
Photopolymerization behavior of the ANR/3D resin blend with various acrylate contents of ANR at the same rubber ratio: (**a**) double-bond (C=C) conversion and (**b**) photopolymerization rate (light intensity = 200 mW/cm^2^ and λ = 405 nm).

Figure 7 shows the double bond conversion and photopolymerization rate of the cured ANR/3D resin blend using a 405 nm UV exposure ranging between 0 and 600 s. The neat commercial 3D resin demonstrated a sharp increment in the maximum conversion of ~87% within approximately 8 s and then slowed down before remaining constant. Theoretically, several factors can affect the conversion efficacy of the radical photopolymerization reaction, including the oxygen inhibition, polymeric chain mobility, and diffusion capacity of active radicals [45,46]. Consequently, it eventually restricts the complete conversion (100%) due to the limitation of polymeric chain motion at the high crosslinking level. The addition of 1.5 wt.% A0.0NR (ANR without acrylate content) did not provide a high conversion, resulting in approximately 60% conversion with the slowest rate of the polymerization reaction, at a cure time of 16 s. However, the utilization of modified NR, with acrylate contents of A0.4NR, A1.5NR, and A3.5NR, suggested a steep increment in the maximum conversion of more than 83% within 10 s. Furthermore, there was a gradual increment in the final conversion and the photopolymerization rate of the ANR/3D resin blend system by utilizing the ANR with a higher acrylate content. These phenomena elucidate the fact that the acrylate moieties on the NR chain could promote the photopolymerization with the 3D resin. At higher quantities of acrylate, the double bond of ANR could probably promote a higher collision between the active unsaturated double bond and the reactive radicals and increase the polymeric chain length or propagating process during the free radical photopolymerization reaction. This result is consistent with the previous literature reported by Chen et al. [47]. They developed high-performance waterborne UV-curable coatings using modified hyperbranched polymers incorporated with water-soluble polyacrylate (WPA) as a crosslinker. As a result, they summarized that an increment of active unsaturated double-bond content could promote the photopolymerization rate and double-bond conversion of the UV-curable product. Moreover, Zhang et al. [48] also found that the prepared polyurethane acrylate resins have a higher acrylate content, suggesting a higher double-bond conversion than the material with lower acrylate moieties.

#### 3.2.3. Mechanical Properties

**Figure 8:**
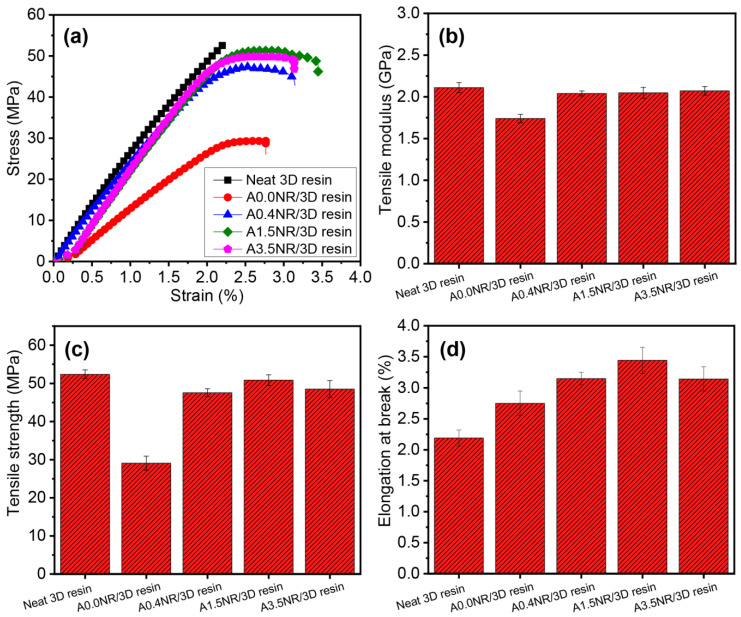
Tensile properties of the printed ANR/3D resin blend with various acrylate contents of ANR at the same rubber ratio: (**a**) stress–strain curves, (**b**) tensile modulus, (**c**) tensile strength, and (**d**) elongation at break.

The prepared liquid resin of rubber/3D resin blends was utilized to fabricate the tensile and impact specimens conforming to the standard of ASTM D638 and ASTM D256, respectively, using the DLP-type 3D printer. Figure 8 shows the tensile properties of the printed ANR/3D resin blends by varying the acrylate contents of ANR at the same weight of rubber content (1.5 wt.%). The neat 3D resin revealed the typical rigid and brittle aspect (Figure 8a). The Young’s modulus and mechanical strength of the neat 3D resin were approximately 2.1 GPa and 52.4 MPa, respectively, with 2.2% elongation ability. The addition of A0.0NR (without acrylate moiety) reduced the Young’s modulus of the printed product from 2.1 GPa (neat commercial 3D resin) down to 1.7 GPa due to the soft characteristics of the added rubber. The utilization of the ANR with acrylate functional group provided a gradual increase in the modulus value from 1.7 GPa (A0.0NR) upward, of approximately 2.1 GPa using ANR containing 3.5 mol% acrylate content (A3.5NR) at the same weight of rubber content (1.5 wt.%). This finding could suggest that the acrylate pendant group attached to the rubber chain may promote the possible interaction between the reactive acrylate functional group of ANR and the reactive radicals of the 3D resin; hence, crosslinking occurred, resulting in increased tensile modulus. This result was consistent with the report of Voet et al. [41]. They attributed an enhancement of the Young’s modulus by increasing the active unsaturated double-bond moiety of the prepared acrylate resin, resulting in the formation of a highly crosslinking network. In this work, the addition of ANR could obviously improve the printed product’s stretchability (Figure 8d), which is the advantage of the soft and elastic characteristics of the NR.

The mechanical strength of the printed A0.0NR/3D resin blend experienced a significant decline to 29.1 MPa, a drop of almost half the strength compared to the neat commercial 3D resin (52.4 MPa). The utilization of ANR with acrylate molecules suggested a gradual increment in mechanical strength up to the maximum value of 50.9 MPa (A1.5NR), but there was a slight decrement to 48.5 MPa (A3.5NR). This result could be attributed to the acrylate reactive sites on the rubber backbone encouraging the chemical interaction between the rubber part and the acrylate-based matrix resin through a radical photopolymerization reaction. The acrylate pendant group on rubber could promote the crosslinking network of the printed material, improving mechanical strength. This aspect was consistent with the report of Anseth et al. [49]. They studied the effect of the double-bond concentration of methacrylate resin on the crosslinking density and the mechanical properties of the cured product. The result found that the higher double-bond concentration of methacrylate resin increased the material’s crosslinking density, mechanical strength, and modulus. In this work, the printed material demonstrated a gradual escalation in stretchability of 2.8% for the printed A0.0NR/3D resin blend, to the maximum value upward of 3.4% for the addition of A1.5NR at the same weight of rubber content. However, the strain at break was slightly dropped to 3.1% when A3.5NR was introduced, but it was still higher than the neat 3D resin. This phenomenon could elucidate the fact that the modified NR containing acrylate functional group could contribute to the partial chemical reaction between the rubber and acrylate-based resin matrix under the radical photopolymerization reaction, improving the stretchability of the printed material at the same ANR content. A higher acrylate content of more than 1.5 mol% could efficiently provide a higher final conversion, as seen in the kinetic profile result in Figure 7, leading to the experiencing of the highly crosslinking network of the bulk material and, hence, it being more brittle [50].

**Figure 9:**
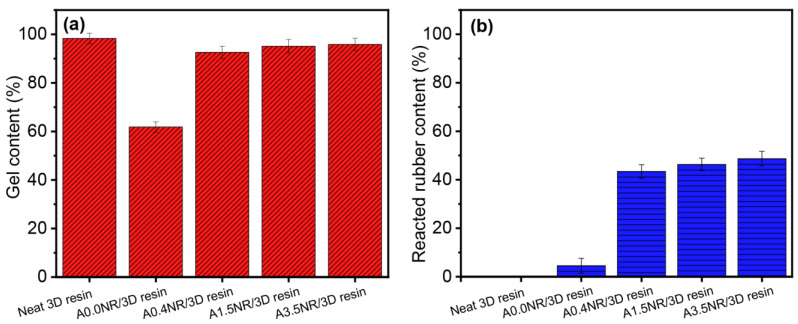
(**a**) Gel content and (**b**) reacted ANR content of the printed ANR/3D resin blend with various acrylate contents of ANR at the same rubber ratio.

The gel content can be related to the crosslinking density of the thermosetting polymer, resulting in the stiffness and mechanical strength of the bulk material [41]. Moreover, the reacted ANR content was investigated to assert the partial interaction of rubber and acrylate-based resin. The gel and the reacted ANR content of the printed rubber/3D resin blend are demonstrated in Figure 9. The neat 3D resin revealed the typical aspect of the highly crosslinking material having a gel content of ~98%. The addition of A0.0NR witnessed a substantial decline in gel content down to ~62%, while the utilization of ANR with acrylate moieties (0.4–3.5 mol%) suggested a significant increased crosslinking density rebounding the gel content ~93% upward. Moreover, there was a gradual elevation in the reacted rubber content when the ANR with the higher amount of acrylate content was introduced to the blend resin system (Figure 9b). It can be noted that there was also a crosslinking reaction that occurred between the rubber chain without acrylate content (A0.0NR) with the 3D resin molecule, probably through the double bond of the NR. As a result, it could be attributed to the fact that the network formation of the modified rubber and 3D resin matrix contributed through not only the acrylate functional groups but also the chain transfer and radical coupling of the double bond of the isoprene unit in the rubber chain during the radical photopolymerization. These phenomena caused an increase in the Young’s modulus, tensile strength, and elongation ability of the printed material [33].

**Figure 10:**
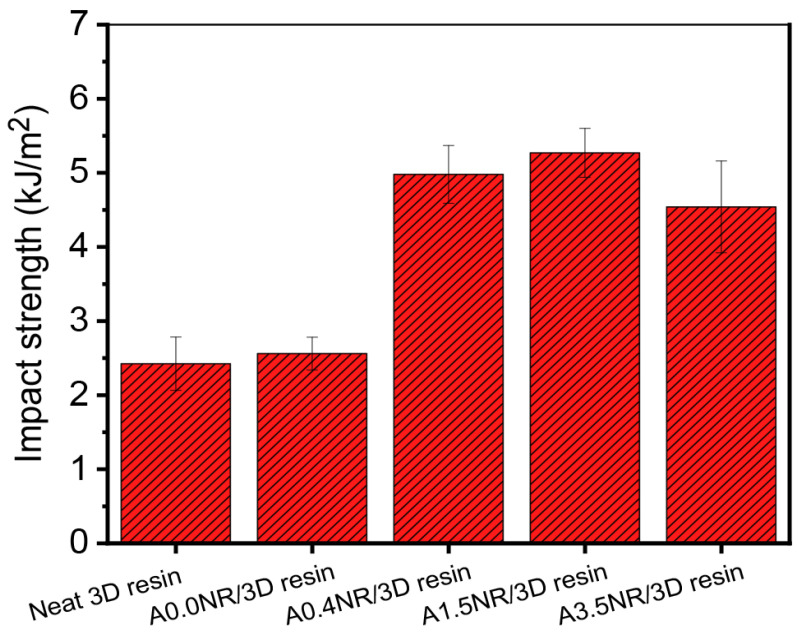
Impact strength of the printed ANR/3D resin blend with various acrylate contents of ANR at the same rubber ratio.

The capability of the printed material to withstand the sudden fracture when the external load is applied, associated with the amount of energy required to fracture a material, is demonstrated by the impact strength value, as illustrated in Figure 10. As a result, there was a slight escalation in the impact strength value from 2.4 kJ/m^2^ (neat 3D resin) to 2.6 kJ/m^2^ by adding 1.5 wt.% of A0.0NR. The presence of ANR with acrylate moieties (A0.4NR, A1.5NR, and A3.5NR) contributed to about a two-fold increase in the impact strength value of the printed product upward to 5.0 kJm^2^ (A0.4NR) and 5.3 kJ/m^2^ (A1.5NR) compared to A0.0NR; however, there was a slight decrement to 4.5 kJ/m^2^ for utilizing A3.5NR. This result could elucidate the fact that the rubber phase can perform as a stress concentrator for absorbing and dissipating energy from an external force, causing a delay in initiating and the propagation of fracture processes. Furthermore, the modified NR with acrylate content could experience a partial interaction with the acrylate-based resin matrix and be more efficient with the higher amount of acrylate content at the same weight ratio of rubber, leading to an increase in the fracture resistance of the material. However, the higher amount of acrylate content of ANR may encourage the higher crosslinking network formation, resulting in an escalation of higher stiffness and, thus, the depletion of the impact strength of the printed product. Additionally, H-bonding between the carbonyl of the acrylate functional groups of the resin and the hydroxyl groups of ANR could also serve as a secondary source, which significantly contributes to the printed materials’ impact resistance [33].

#### 3.2.4. Morphological Properties

**Figure 11:**
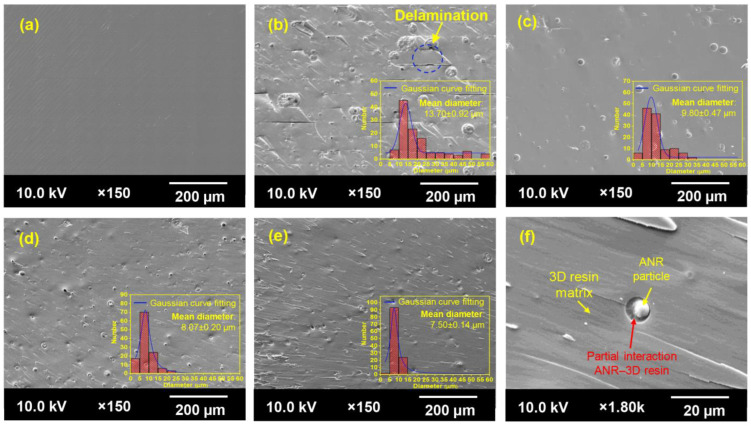
SEM images of the impact-fractured surface of printed ANR/3D resin blend with various acrylate contents of ANR at the same rubber ratio: (**a**) neat 3D resin, (**b**) A0.0NR/3D resin, (**c**) A0.4NR/3D resin, (**d**) A1.5NR/3D resin, (**e**) A3.5NR/3D resin, and (**f**) A3.5NR/3D resin at ×1.80 k magnification.

The phase morphological aspect of the printed samples was observed using scanning electron microscopy (SEM) analysis, as illustrated in Figure 11. Generally, the 3D-printed part’s mechanical performance can be associated with phase morphologies. Achieving a well uniform dispersion and distribution of the second phase in the polymer blend system is critical to significantly enhance the mechanical performance of the desired material [51]. As a result, the printed neat 3D resin revealed a smooth and homogeneous fashion (Figure 11a). The addition of all types of ANR exhibited the inhomogeneous surface system with dispersed ANR particles as a second phase in the 3D resin matrix (Figure 11b–e). This result can be attributed to the non-equilibrium thermodynamics between the ANR phase and acrylate resin matrix because of the complexity of the low-diffusion ability and the polymer chain mobility via the radical photopolymerization reaction during the 3D printing process. The dispersed rubber particles could perform as a stress concentrator to retard the initiation and propagation of the cracking process after an external load was applied [25].

Additionally, there was an apparent decrement in the dispersed rubber particles from ~14 µm for the utilization of A0.0NR gradually down to ~8 µm for the addition of A3.5NR. This phenomenon could elucidate the probably partial interaction of the rubber phase and acrylate resin matrix through the radical photopolymerization reaction, being a decrease in the dispersed rubber particles in the resin matrix. Moreover, the phase morphology of A3.5NR/3D resin (Figure 11f) at higher magnification (×1.80 k) was selected from the red dot rectangular line of Figure 11e. As a result, the phase interaction between the dispersed rubber particle and acrylate 3D resin matrix was observed. This finding is consistent with the report of Chiappone et al. [52]. They investigated the morphologies of the 3D-printed parts of poly(ethylene glycol) diacrylate-based hybrid nanocomposites and explored the formation of finer droplets in the acrylate matrix by reducing the interfacial tension. Regarding the phase morphology of the printed A0.0NR/3D resin blend, it observed the agglomeration of rubber and the delamination defect on the fractured surface. This result could suggest a poor interaction between the rubber phase and the 3D resin matrix, causing a significant deterioration in mechanical strength and stretchability, as aforementioned.

#### 3.2.5. Thermal Properties

**Figure 12:**
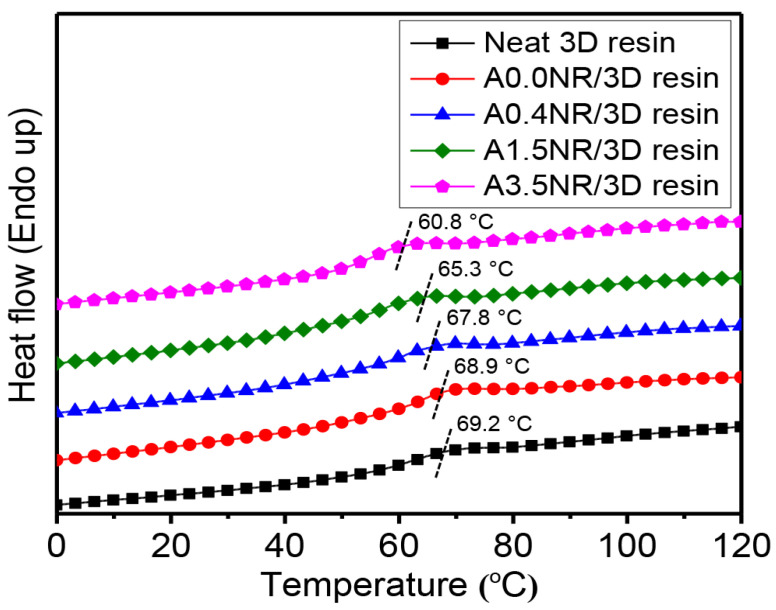
The 2nd DSC thermograms of the printed ANR/3D resin blend with various acrylate contents of ANR at the same rubber ratio.

Generally, the glass transition temperature (T_g_) can be related to the segmental relaxation (α-relaxation process), dependent upon the entanglement and the crosslinking density [53], and the interfacial interaction [54,55] of the desired polymeric material. Therefore, the higher entanglement and crosslinking density can provide the material with a higher T_g_ value. On the other hand, the improving interfacial interaction between two or more components and the matrix can provide a lowered T_g_ of the bulk material. The T_g_ values of the printed ANR/3D resin blends with various acrylate contents of ANR at the same rubber ratio were measured using differential scanning calorimetry (DSC) analysis, as shown in Figure 12. As a result, the printed neat 3D resin revealed the glass transition relaxation between 60 and 70 °C and centered at 69.2 °C. The addition of 1.5 wt.% of A0.0NR (ANR without acrylate group) did not significantly influence the segmental relaxation of the resin matrix, having a T_g_ of 68.9 °C. This result is not consistent with the conversion and the gel behavior of the printed A0.0NR/3D resin blend, as aforementioned. It may be suggested that there was an increment in crosslinking density from the thermal treatment during the heat-cool-heat cycles of the DSC measurement. In the cases of the utilization of ANR having various acrylate contents at the same rubber ratio, it witnessed a gradual shift in the T_g_ value of the printed product downward towards the lower temperature zone at 67.8 °C for A0.4NR/3D resin, which further lowered to 60.8 °C for A3.5NR/3D resin. This phenomenon could be attributed to a partial linkage formation between the rubber phase (lower T_g_ material) and acrylate resin matrix (higher T_g_) under the radical photopolymerization reaction during the lithographic 3D printing process, resulting in the lowered T_g_ of the matrix resin.

## 4. Conclusions

The prepared low-molecular-weight natural rubber with various acrylate contents (0.0–3.5 mol%), as a photosensitive mechanical modifier for the lithographic 3D printing process, was achieved via the three steps of chemical modifications using NR latex as a raw material. The addition of ANR with acrylate content (0.0–3.5 mol%) to the commercial 3D resin using a simple mixing method could escalate the viscosity of the mixture resin system without altering its rheological manners. The mechanical properties of the printed sample were improved by increasing the acrylate content in the ANR. The ANR with 1.5 mol% acrylate content (A1.5NR) could provide the maximum improvement in the stretchability and impact strength, approximately 155% and 221%, respectively, compared to those of the neat 3D resin without the significant deterioration of the tensile modulus and mechanical strength, as well as the crosslinking density and photopolymerization rate. The printed ANR/3D resin showed a heterogeneous surface with small rubber particles dispersed in the 3D resin matrix. The partial interaction of dispersed rubber particles and resin matrix could observe from the reduction in rubber particles’ diameter and the shift of T_g_ of the printed sample to the lower temperature by utilizing ANR having higher acrylate content. Therefore, this research work could provide a convenient way to improve the basic commercial 3D resin’s properties and extend the widespread end-use applications by incorporating the prepared photosensitive NR. Notably, it suggests a new application for natural rubber as a bio-based material in lithographic 3D printing technology.

## Figures and Tables

**Table 1:** Blend composition of ANR/3D resin blend.

Sample Code	Acrylate Content in ANR (mol%)	Blend Composition	
		ANR Solution (wt.%)	3D Resin Content (wt.%)
Neat 3D resin	-	0	100
A0.0NR/3D resin	0.0	5	95
A0.4NR/3D resin	0.4	5	95
A1.5NR/3D resin	1.5	5	95
A3.5NR/3D resin	3.5	5	95

**Table 2:** Molecular weight and polydispersity index of NR and the modified NRs.

Sample	M_n_ (g/mol)	M_w_ (g/mol)	PDI
NR	908,195 ± 23,391	1,184,513 ± 218,341	1.34 ± 0.14
A0.0NR	14,441 ± 3703	47,132 ± 3309	3.26 ± 0.12
A0.4NR	14,532 ± 2118	46,364 ± 2940	3.22 ± 0.18
A1.5NR	14,615 ± 3100	45,752 ± 4331	3.14 ± 0.20
A3.5NR	14,387 ± 2860	46,303 ± 3710	3.21 ± 0.25

## Data Availability

The data presented in this study are available on request from the corresponding author.
